# Full-thickness skin graft versus split-thickness skin graft for fasciocutaneous radial forearm free flap donor site closure: a systematic review and meta-analysis

**DOI:** 10.1186/s13643-025-02863-7

**Published:** 2025-05-27

**Authors:** Jasper J. E. Moors, Zhibin Xu, Kunpeng Xie, Ashkan Rashad, Oliver Vladu, Jan Egger, Rainer Röhrig, Frank Hölzle, Behrus Puladi

**Affiliations:** 1 Department of Oral and Maxillofacial Surgery, University Hospital RWTH Aachen, Aachen, 52074 Germany; 2 Institute of Medical Informatics, University Hospital RWTH Aachen, 52074 Aachen, Germany; 3 Cancer Research Center Cologne Essen (CCCE), West German Cancer Center Essen (WTZ), 45122 Essen, Germany; 4 Institute of Artificial Intelligence in Medicine, Essen University Hospital, 45131 Essen, Germany

**Keywords:** Radial forearm free flap, Donor site closure, Donor site morbidity, Split-thickness skin graft, Full-thickness skin graft

## Abstract

**Background:**

The radial forearm free flap (RFFF) is widely used in microvascular reconstructions. However, donor site morbidity remains a concern, with complications such as wound healing issues, functional impairments, and aesthetic concerns. While both full-thickness skin grafts (FTSG) and split-thickness skin grafts (STSG) are commonly used for donor site closure, there is insufficient evidence to determine which technique leads to fewer complications. This study aims to systematically compare FTSG and STSG in RFFF donor site closure.

**Methods:**

We searched six databases and four clinical trial registries up to 1 March 2025. We focused on studies comparing FTSG and STSG. Primary outcome was the incidence of wound complications. Secondary outcomes included functional and aesthetic impairment. Risk of bias was assessed using the Risk Of Bias In Non-Randomized Studies—of Interventions (ROBINS-I) and quality of the evidence using the Grading of Recommendations Assessment, Development and Evaluation (GRADE) approach.

**Results:**

Fifteen studies were analyzed, involving 933 donor site closures. No RCTs met our inclusion criteria. Meta-analysis comparing FTSG versus STSG revealed no significant differences in major wound complications (*RR* 0.43; 95% *CI* 0.11 to 1.70; *p* = 0.23) and minor wound healing complications (*RR* 0.83; 95% *CI* 0.60 to 1.13; *p* = 0.23), with the evidence graded as low to very low certainty.

**Conclusion:**

Current evidence does not conclusively favor either FTSG or STSG for radial forearm free flap donor site closure regarding wound, functional, or aesthetic outcomes. Future well-designed RCTs are needed to provide higher-quality evidence to guide clinical decision-making. Until more robust evidence becomes available, the optimal skin graft choice should be guided by patient-specific factors, surgical considerations, and donor site characteristics.

**Systematic review registration:**

PROSPERO CRD42023351903.

**Supplementary Information:**

The online version contains supplementary material available at 10.1186/s13643-025-02863-7.

## Background

The radial forearm free flap (RFFF) was first described by Yang et al. in 1981 as a fasciocutaneous flap [1]. Due to its relative thinness, pliability, long and high-caliber pedicle, and reliable anatomy, it has been established as a workhorse in microvascular reconstructive surgery [2, 3] and employed in a diverse array of reconstruction purposes, including head and neck reconstruction, limb reconstruction, and phalloplasty [1, 4]. The flap is harvested from the volar aspect of the nondominant forearm and consists of the skin, subcutaneous tissue, and the forearm fascia, with the radial artery and two concomitant veins forming the vascular pedicle [5]. After flap raising, the donor site is usually closed by using a split-thickness skin graft (STSG), as originally proposed by Yang et al. [1], or by using a full-thickness skin graft (FTSG) [6]. However, RFFF harvesting could potentially lead to donor site morbidity, such as tendon exposure, altered sensitivity, and impaired arm and hand function [4, 7–9].

As surgical advancements have increased recipient outcomes, minimizing donor site morbidity has become a critical objective [10, 11]. Different wound closure techniques and flap modifications have been explored to decrease donor site morbidity, including the use of local FTSGs to avoid having a third surgical site [12–27], suprafascial flap raising (cutaneous RFFF) [28–30], the snake flap design [31], the prefabricated split skin fascia flap [32], preoperative tissue expansion in order to primarily close the defect [33–39], local flaps to primarily close the defect [26, 40–45], and the use of dermal substitutes [46–54]. Other refinements that have been suggested include the creation of a well-vascularized wound bed by mobilization of adjacent muscles [9, 55–58], quilting of the skin graft [55, 59], and negative pressure wound therapy instead of conventional bolster dressing [60, 61]. Ideally, with a small donor site, primary wound closure can be attempted, but this is less common. Methods like purse string suturing [19, 62–64] and cross suturing [65] have been applied in order to reduce the size of the defect. Despite these versatile closure techniques, the majority of surgeons report RFFF donor site closure using FTSGs (50%) and STSGs (40%), making these the most common closure techniques [9, 66].

Nevertheless, a definitive consensus on the best closure method (FTSG vs. STSG) for RFFF remains elusive due to conflicting outcomes from controlled studies. This ambiguity underscores the necessity of evidence-based surgery. To date, there has been no systematic review that exclusively summarizes the evidence on donor site closure for fasciocutaneous RFFF. Although not directly transferable to the RFFF alone, three recent systematic reviews of the RFFF/osteocutaneous radial forearm flap (OCRFFF) reached conflicting conclusions on this issue [67–69]. The focus on both fasciocutaneous RFFF and OCRFFF patients creates confusion because OCRFFF is significantly more invasive than fasciocutaneous RFFF due to bone harvesting and the risk of radial fracture [9]. Moreover, OCRFFFs are seldomly used for head and neck reconstruction as the main options for osseous reconstructions are considered the fibula free flap, deep circumflex iliac artery flap, and scapula free flap [70–72]. Outside of the head and neck, it is used primarily for hand and thumb reconstruction [73]. Further complicating the interpretation of existing evidence, these systematic reviews on RFFF/OCRFFF have several shortcomings, such as not using comprehensive risk-of-bias assessments or not including all relevant papers. This evidence makes extrapolating the potential conclusions of donor site closure to the fasciocutaneous RFFF alone even more difficult. Furthermore, although the RFFF originated in China, and Chinese surgeons have made significant contributions to microvascular flap surgery, none of the authors included Chinese literature or databases in their search [74].

Our study is the first systematic review and meta-analysis focusing solely on fasciocutaneous RFFF and aims to answer the question of whether, in patients requiring fasciocutaneous RFFF surgery, donor site closure using an FTSG compared to an STSG leads to improved wound-related, functional, and aesthetic outcomes. The insights gained could significantly influence clinical practice and play a crucial role in developing future clinical guidelines.

## Methods

This systematic review and meta-analysis was conducted according to our study protocol published in this journal *Systematic Reviews* [75] and reported in accordance with the Preferred Reporting Items for Systematic reviews and Meta-Analyses (PRISMA) 2020 statement [76]. The study protocol was registered with the International Prospective Register of Systematic Reviews (PROSPERO) on 17 September 2023 (registration number CRD42023351903) and updated on 4 March 2024 after publication of the protocol.

### Eligibility criteria

We applied the PICO framework (Table 1) alongside a predefined list of inclusion and exclusion criteria (Table 2). Our inclusion criteria focused on studies published in peer-reviewed journals that directly compared outcomes between FTSG and STSG closures in adult human patients. We excluded animal studies, cadaveric studies, and research focusing on OCRFFF patients. The main intervention was surgical closure of the RFFF donor site with FTSG, compared against closures with STSG, following the initial proposal by Yang et al. [1]. Studies incorporating STSG with dermal substitutes versus FTSG alone were not considered to ensure clarity in the comparison [77–79]*.* Our analysis focused on wound, functional, and aesthetic outcomes (Table 3).

**Table 1:** PICO statement

P (population)	Patients with tissue defects in the maxillofacial, limb, or genital region requiring reconstruction with a fasciocutaneous RFFF
I (intervention)	Surgical closure using FTSG
C (comparison)	Surgical closure using STSG
O (outcome)	Wound-, functional-, and aesthetics-related outcomes

**Table 2:** Inclusion and exclusion criteria

Inclusion criteria	Studies comparing RFFF donor site closure with FTSG versus RFFF donor site closure with STSG
	RCTs, prospective and retrospective comparative cohort studies
	Patients ≥ 18 years
	Follow-up ≥ 1 month for wound-related and function-related outcome measures Follow-up ≥ 3 months for aesthetics-related outcome measures Studies in English, German, and Chinese language Articles from 1981 and younger
Exclusion criteria	Cadaveric and animal studies
	Studies regarding osseous RFFF

**Table 3:** Definition of outcomes

Outcome	Measures
Wound related	Tendon exposure^a^
	Dehiscence^a^
	Complete graft loss^a^
	Partial graft loss^b^
	Hematoma^b^
	Seroma^b^
	Delayed healing^b^
	Need for redressing^b^
	Infection^b^
Function related	Pain
	Sensory deficits
	Decreased range of motion (ROM)
	Decreased strength
	Disability of Arm, Shoulder and Hand (DASH) [80]
	Mayo wrist score [81]
	Cold Intolerance Severity Score (CISS) [82]
Aesthetic related	Coloration
	Thickness
	Scarring
	Patient and Observer Scar Assessment Scale (POSAS) [83]

^a^Major wound complication

^b^Minor wound complication

### Search strategy

We searched six databases: PubMed/MEDLINE, Embase, Scopus, Web of Science, the Cochrane Central Register of Controlled Trials (CENTRAL), and China National Knowledge Infrastructure (CNKI) and four clinical trial registers: ClinicalTrials.gov (clinicaltrials.gov), the German Clinical Trials Register (www.drks.de), the ISRCTN registry (www.isrctn.com), and the International Clinical Trials Registry Platform (trialsearch.who.int). The search was limited to publications from January 1981 onward (after the first description of the RFFF) and was updated until 1 March 2025. The search query for PubMed is listed in Table 4, and search queries for all databases and search platforms are listed in Additional file 1.

**Table 4:** Search strategy for PubMed

Database	Search query
PubMed	(("Surgical Flaps"[MeSH Terms] OR"Surgical Flap*"[Title/Abstract] OR"flap surgical*"[Title/Abstract] OR"flaps surgical*"[Title/Abstract] OR"radial forearm flap*"[Title/Abstract] OR"radial forearm free flap*"[Title/Abstract]) AND ("Skin Transplantation"[MeSH Terms] OR"Skin Transplantation*"[Title/Abstract] OR"transplantation skin*"[Title/Abstract] OR"grafting skin*"[Title/Abstract] OR"skin graft*"[Title/Abstract] OR"dermatoplast*"[Title/Abstract]) AND ("Forearm"[MeSH Terms] OR"Forearm*"[Title/Abstract] OR"radial*"[Title/Abstract] OR"antebrachi*"[Title/Abstract])) AND (1981:2025/03/01[pdat])

### Data collection

**Fig. 1:**
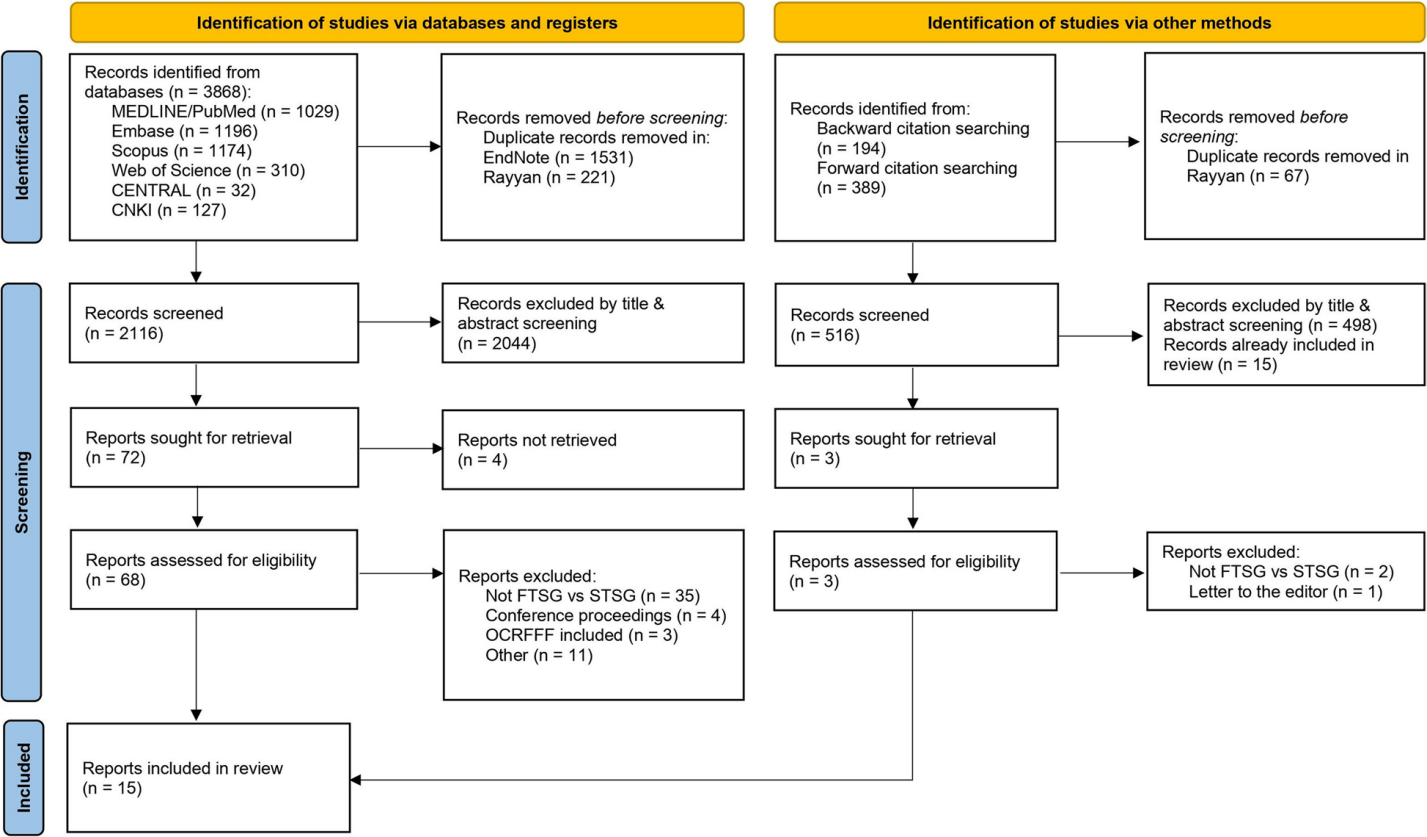
PRISMA flow diagram summarizing the study selection process

A total of three reviewers (English and German: J. M. and Z. X; Chinese: Z. X. and K. X.) performed title and abstract screening in a blinded manner using the tool Rayyan.ai. All data that two reviewers could not clearly exclude based on its title and abstract received a full-text review. A study was included when two reviewers independently assessed it as satisfying the inclusion criteria from the full text. If a disagreement remained after discussion, the fourth reviewer (B. P.) mediated. Reasons for excluding trials were recorded. After completion of the selection process, a PRISMA flow diagram was created (Fig. 1).

The same reviewer pairs performed data extraction in a blinded manner, and the fourth reviewer mediated if necessary. We contacted the authors of the included studies [84, 85] through e-mail in case of uncertainties, with a maximum of three attempts. Unfortunately, none of these attempts was successful.

### Outcomes and prioritization

#### Primary outcomes

Wound outcomes were considered primary outcomes and divided into major and minor wound complications (Table 3). Major wound complications included tendon exposure, dehiscence, and complete graft loss, whereas minor wound complications included partial graft loss, hematoma, seroma, delayed healing, need for redressing, and infection.

#### Secondary outcomes

Functional and aesthetic outcomes were considered secondary outcomes (Table 3). Functional outcomes included pain, sensory deficits, decreased range of motion (ROM), decreased strength, Disability of Arm, Shoulder and Hand (DASH) [80], the Mayo wrist score [81], and the Cold Intolerance Severity Score (CISS) [82]. Aesthetic outcomes included coloration, thickness, scarring, and the Patient and Observer Scar Assessment Scale (POSAS) [83].

### Data items

Variables for which data were collected were study characteristics (study design, country, start date, end date, number of participants, number of procedures, time to follow up, source of financial support), patient characteristics (mean age, sex, indication for surgical procedure), and intervention details (donor site defect size in cm^2^ and flap raising technique {i.e., suprafascial vs. subfascial}).

### Risk of bias in individual studies

Risk of bias was assessed for each included study and per outcome domain by the same reviewer pairs using the Risk Of Bias In Non-Randomized Studies—of Interventions (ROBINS-I) tool [86]. The results of the risk of bias assessment were visualized using *robvis* [87].

### Data synthesis and statistical methods

Meta-analyses were performed using Review Manager (RevMan) 5.4.1. The potential inclusion of other study types alongside RCTs was determined using a decision tree adapted from the Cochrane algorithm and is further detailed in the study protocol [75]. We collected dichotomous data on major and minor wound outcomes, specifically assessing the occurrence of wound complications as present or absent. For statistical analysis, we calculated relative risk (RR) with a 95% confidence interval (CI) to compare the incidence of wound complications between the treatment groups.

To evaluate between study variance, the *I*^2^ index was used with values of 25%, 50%, and 75% representing low, moderate, and high heterogeneity, respectively [88]. Between-study variance was expected to be present as studies from different centers across the world with different wound care protocols, and different suturing techniques and materials were included. Given the initial expectation of high heterogeneity, a random-effects model for meta-analysis regarding both major and minor wound complications was considered. However, statistical analysis of studies comparing minor wound complications revealed low heterogeneity (*I*^2^ = 0%). Therefore, a fixed-effects model was used for the primary analysis.

### GRADE

The certainty of evidence for outcomes was assessed using the Grading of Recommendations Assessment, Development and Evaluation (GRADE) approach [89]. Summary of findings (SoF) tables and a GRADE evidence profile were created (Additional file 2).

## Results

### Study selection

The search resulted in 3980 records. After duplicate removal, we screened 2182 records from which we excluded 2108 records based on title and abstract. Seventy-four records remained for full-text review, from which we included 15 publications [13, 16, 23, 30, 58, 84, 85, 90–97]. Backward citation searching and forward citation searching using citationchaser [98] for included studies did not yield any additional studies that fulfilled the eligibility criteria (Additional file 3).

One study was suspected to have overlapping data with another study from the same author [84, 99]. Since we were not able to reach the author, we opted to exclude the study with the fewest number of cases from our analysis to prevent redundancy and ensure the inclusion of unique data [99].

### Excluded studies and justification

Studies that might appear to meet the inclusion criteria but were excluded from this systematic review were studies by Burger et al. [78], Di Giuli et al. [77], and Watfa et al. [79] These studies were excluded due to methodological concerns. Each study compared STSG in combination with a dermal substitute against FTSG alone, lacking a direct comparison and potentially introducing bias. Furthermore, Avery et al. [100] and Sidebottom et al. [59] were excluded because of the inclusion of both patients with fasciocutaneous as well as patients with OCRFFF flaps. Davis et al. [101] were excluded because they included both free and pedicled radial forearm flaps. The pedicled radial forearm flap is generally harvested more proximally on the forearm than the RFFF and therefore exposes muscle bellies rather than tendons [102]. Hence, we consider it a different type of flap.

### Study characteristics

The 15 included studies included 10 retrospective cohort studies [16, 23, 58, 85, 90–93, 96, 97] and 5 prospective cohort studies [13, 30, 84, 94, 95] (Table 5). There were no RCTs that met the eligibility criteria. Twelve of these studies had a two-armed design (FTSG vs. STSG), and 3 of these studies had a three-armed design, in which one treatment arm used a combination of a skin graft with a dermal substitute.

**Table 5:** Study characteristics

Author and year	Study design	Country	Patients	FTSG vs. STSG					Period	Indication	Technique	Outcome		
				No. of procedures	Age (years)	Sex	Donor site defect size (cm^2^)	Follow-up (months)				Wound	Function	Aesthetics
Al-Aroomi, 2023 [96]	Retrospective cohort study	China	75	35/40	54.6 ± 9.9/56.7 ± 9.5	13F + 22M/11F + 29M	40 ± 6.2/41.4 ± 7.0	22.5 ± 12.8/12.0 ± 2.9	3/2017–8/2021	H&N	Subfascial	Yes	Yes	Yes
Avery, 2007 [30]	Prospective cohort study	UK	120	94/27	57 ± 14	43F + 57M	35 (11–96)/72 (36–126)	15 (4–49)/19 (4–36)	3/1999–1/2006	H&N (17), limb (8)	Suprafascial	Yes	No	No
Bonaparte, 2013 [94]	Prospective cohort study	Canada	177	120/8	65.6 ± 12.1/56.0 ± 21.1	57F + 63M/1F + 7M	38.6 ± 12.9/44.3 ± 16.9	Repeatedly, minimum 1 year	1/2008–9/2011	H&N	Subfascial 87% (not further specified)	Yes	No	No
Chambers, 1997 [13]	Prospective cohort study	UK	21	16/5	NR	NR	NR	≥ 3	NR	H&N	Subfascial	Yes	Yes	Yes
Cristofari, 2020 [85]	Retrospective cohort study	France	43	15/14	44 (25–77)/41 (22–76)	4 + 4F + 7M/6 + 5F + 3M	12–14 (length)/11–16 (length)	24 (11–30)/28 (14–34)	1/2009–4/2014	H&N, Limb, phalloplasty	Suprafascial	Yes	Yes	Yes
Ho 2006, [97]	Retrospective cohort study	USA	25	8/10	54 (40–74)/66 (32–79)	2F + 6M/3F + 7M	NR	8 (3–12)/51 (27–78)	7 years	H&N	NR	No	Yes	Yes
Krane, 2020 [90]	Retrospective cohort study	USA	136	68/68	65.9 ± 10/63 ± 15	24F + 44M/29F + 39M	69.2 ± 27.4 (12–144)/56.7 ± 31.8 (16.5–170)	6.6 ± 5.1/26.5 ± 29.9	FTSG: 4/2016–11/2017; STSG: 1/2009–5/2010	NR	Subfascial	Yes	Very limited	Yes
Lee, 2019 [95]	Prospective cohort study	South Korea	20	10/10	61/57.2	1 F + 9M +/1F + 9M	60 (40–77)/92 (48–126)	6	7/2014–8/2018	H&N	NR	Yes	No	Yes
Lutz, 1999 [84]	Prospective cohort study	Taiwan	95	31/64	50.1 (26–83)	6F + 89M	78.3 (24–191)	Wound: during admission/function and aesthetics: ≥ 6	3/1995–11/1997	H&N	Suprafascial	Yes	Yes	Yes
Molteni, 2022 [92]	Retrospective cohort study	Italy	36	19/17	57/63	7F + 12M/10F + 7F	NR	≥ 6	2/2016–9/2020	Reconstructive surgery	Subfascial	No	Yes	Yes
Peters, 2021 [91]	Retrospective cohort study	Germany	30	15/15	64.8 (38–80)/64.8 (44–80)	7F + 8M/8F + 7M	NR	≥ 6	NR	H&N	Subfascial	No	No	Yes
Selvaggi, 2006 [58]	Retrospective cohort study	Belgium	125	47/78	NR	125F to M	228	43 (6–108)	11/1993–10/2003	Phalloplasty	74 subfascial, 19 suprafascial, 32 combination	Yes	Yes	Yes
Thiele, 2008 [93]	Retrospective cohort study	Germany	25	17/8	NR	NR	NR	3 (3–108)	7/1996–7/2006	H&N	Suprafascial	Very limited	Yes	Yes
Vahldieck, 2022 [23]	Retrospective cohort study	Germany	40	21/19	60 ± 15.25/65 ± 18.0	9F + 12M/12F + 7M	27.3 ± 9.78/27.1 ± 8.33	≥ 3	1/2020–1/2021	H&N	Suprafascial	Very limited	Yes	Yes
Zuidam, 2005 [16]	Retrospective cohort study	The Netherlands	34	19/15	62.0 ± 9.0/56.8 ± 10.2	6F + 13M/7F + 8M	26.2 (20–40)/40.4 (15–96)	25.3 ± 15 (4.3–50.7)/26.1 ± 9.3 (11.3–35.9)	1/2001–6/2004	H&N	Suprafascial	Very limited	Yes	Yes

Abbreviations: *NR* Not reported, *F* Female, *M* Male, *H&N* Head and neck surgery, *Limb* Limb reconstruction

Across the 15 included studies, a combined total of 933 donor site closures were performed, comprising 535 procedures utilizing FTSGs and 398 procedures involving STSGs. Reported age of treated patients ranged from 25 to 77 years in the FTSG group and from 22 to 79 years in the STSG group. Reported mean defect size ranged from 26.2 to 69.2 cm^2^ in the FTSG group and from 27.1 to 56.7 cm^2^ in the STSG group. Studies were published between 1997 and 2023, and the indications included head and neck reconstruction, limb reconstruction, and phalloplasty.

### Risk of bias in studies

**Fig. 2:**
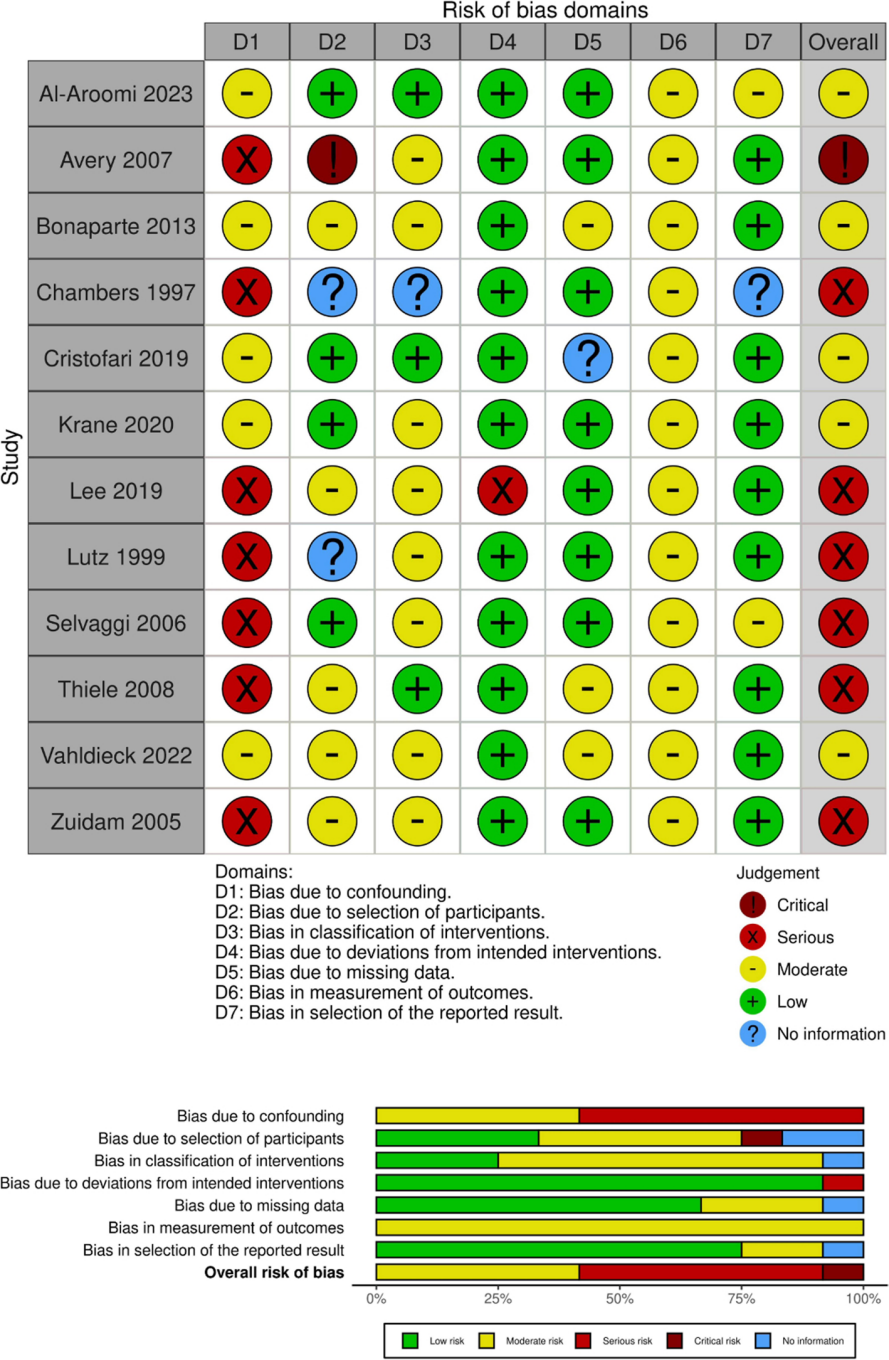
Risk-of-bias assessment (ROBINS-I) for studies reporting on wound outcome

**Fig. 3:**
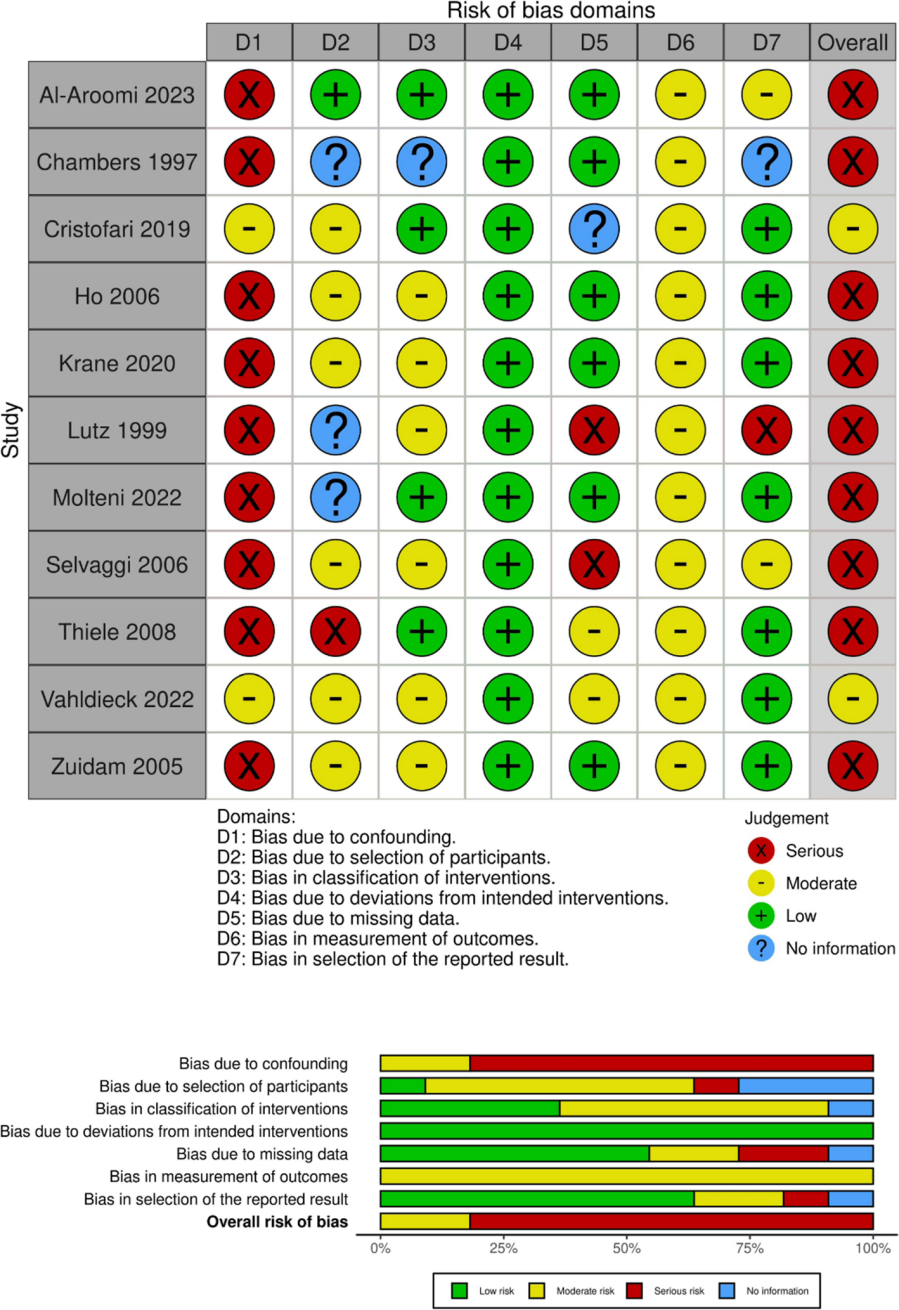
Risk-of-bias assessment (ROBINS-I) for studies reporting on functional outcome

**Fig. 4:**
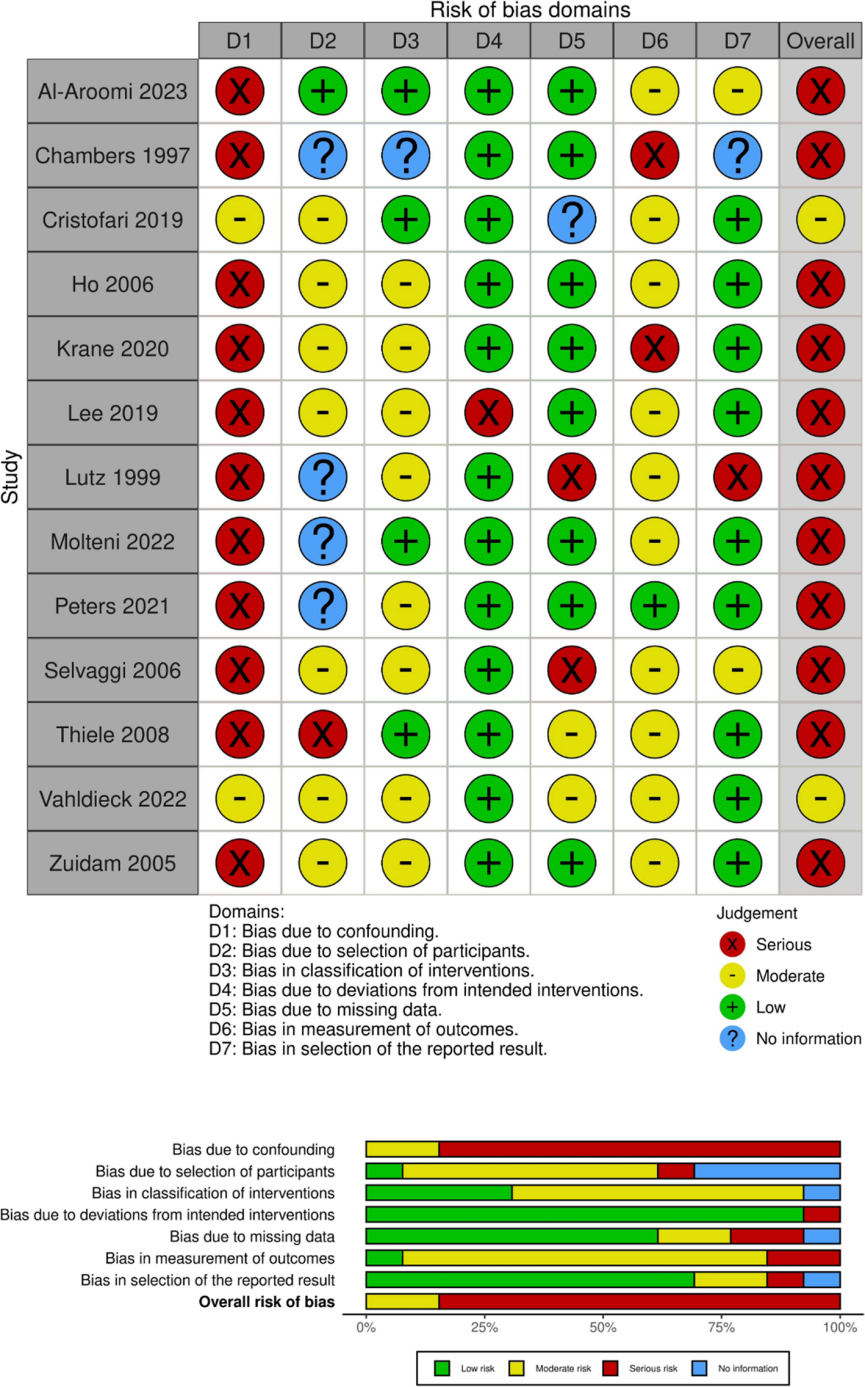
Risk-of-bias assessment (ROBINS-I) for studies reporting on aesthetic outcome

The results of the risk-of-bias assessment for each included study and per outcome domain are displayed in Figs. 2, 3, and 4. The majority of studies showed a serious risk of bias due to confounding, mostly because of not reporting donor site defect size or time to follow-up or because they did not correct for a significant difference in patient characteristics between groups. Among the 12 studies reporting wound outcomes, 5 studies exhibited a moderate risk of bias [23, 85, 90, 94, 96], 6 studies exhibited a serious risk of bias [13, 16, 58, 84, 93, 95], and 1 study was identified as having a critical risk of bias [30] (Fig. 2). Among the 11 studies reporting function-related outcomes, 2 studies exhibited a moderate risk of bias [23, 85], whereas 9 studies exhibited a serious risk of bias [13, 16, 58, 84, 90, 92, 93, 96, 97] (Fig. 3). Among the 13 studies reporting aesthetic outcomes, 2 studies had a moderate risk of bias [23, 85], and 11 studies showed a serious risk of bias [13, 16, 58, 84, 90–93, 95–97] (Fig. 4).

### Results of individual studies

#### Wound-related outcomes

Twelve studies with a total of 493 donor site closures using FTSG and 356 donor site closures using STSG were reported on wound outcome. Al-Aroomi et al. [96] observed no statistically significant differences in edema, skin graft loss, or tendon exposure between the two groups. Avery et al. [30] reported a significantly shorter median time to healing for FTSG than for STSG (14 compared to 10 days). Bonaparte et al. [94] also reported a significantly faster time to healing in the FTSG group as well (8.7 days compared to 13.6 days mean time to healing). The authors reported two cases of tendon exposure in the STSG group compared to two cases of complete graft loss and two cases of partial graft loss in the FTSG group but did not perform statistical analysis. Chambers et al. observed no events of skin loss or tendon exposure in any of the treatment groups [13]. Cristofari et al. [85] reported cases of hematoma, infection, and partial graft necrosis in both treatment groups but found no significant differences. Krane et al. [90] found no statistical differences between groups regarding graft loss, tendon exposure, infection, and hematoma/seroma. Vahldieck et al. [23] detected neither major nor minor wound complications in either of the treatment groups. Lee et al. observed faster healing in the FTSG group than in the STSG group, but the difference was not significant [95]. Lutz et al. [84], Selvaggi et al. [58], Thiele et al. [93], and Zuidam et al. [16] reported their complications without statistical testing. Lutz et al. [84] and Selvaggi et al. [58] reported a higher skin graft take rate for STSG, Thiele et al. [93] reported one case of wound healing failure in the STSG group compared to zero cases in the FTSG group, and Zuidam et al. [16] reported one case of partial graft necrosis in the STSG group, compared to none in the FTSG group.

#### Function-related outcomes

Eleven studies with a total of 296 donor site closures using FTSG and 338 donor site closures using STSG were reported on function-related outcome. Al-Aroomi et al. [96] reported a significantly better grip strength and range of wrist movement in favor of STSG. In contrast, the authors observed a significantly better outcome regarding cold intolerance in favor of the FTSG. Chambers et al. [13] evaluated sensation, pain, and grip strength without statistical testing. Cristofari et al. [85] assessed functional outcome using the DASH score. The FTSG group had a significantly improved DASH score (10.6/100) compared to the STSG group (16.7/100). Ho et al. [97] found an improved ROM in STSG patients. Krane et al. [90] saw no difference in subjective functional impairment. Lutz et al. [84] reported their results not specifically for FTSG vs. STSG, limiting their suitability for analysis. Molteni et al. [92] measured no significant difference in patients’ subjective satisfaction. Selvaggi et al. [58] evaluated pressure and vibratory thresholds and observed a higher pressure threshold in FTSG forearms, but did not perform statistical testing. Thiele et al. [93] reported a greater incidence of paresthesia, loss of sensation, and heat and cold intolerance in the STSG group, but also did not perform statistical testing. Vahldieck et al. [23] assessed functional outcomes evaluating active ROM deficit, hand grip strength, and hypoesthesia but no significant differences were found between the treatment groups. Additionally, their patient survey, which evaluated subjective limitations in mobility, strength dexterity, and sensitivity in the fingers, hand, and forearm, revealed no significant differences. Zuidam et al. [16] measured DASH score, sensibility, active ROM of the hand, and grip strength and reported no significant differences [103].

#### Aesthetic-related outcomes

Thirteen studies with a total of 321 donor site closures using FTSG and 363 donor site closures using STSG were reported on aesthetics-related outcome. Al-Aroomi et al. [96] investigated the aesthetic appearance of the donor site by using a 4-item scale ranging from very good to poor. The overall aesthetic appearance was assessed as being significantly better in the FTSG group but only when assessed by the patient. Chambers et al. [13] assessed aesthetic appearance using a 3-item scale ranging from excellent to poor without statistical testing. Cristofari et al. [85] evaluated aesthetic outcome using the Vancouver Scar Scale (VSS) and a custom-made satisfaction scale for both patients and surgeons. No significant differences were reported. Ho et al. [97] reported no significant differences between treatment groups when evaluating aesthetic outcome by a panel of head and neck surgeons. Krane et al. [90] evaluated aesthetic outcome using a 5-item scale. Aesthetic outcome was superior in the FTSG group when assessed by both the patient and the surgeon. Lee et al. [104] investigated donor sites using the VSS and reported that compared with the STSG, the FTSG was significantly better in terms of pigmentation, pliability, and height. Lutz et al. [84] evaluated aesthetic satisfaction with a 0 to 10 rating by both the patient and an investigator, but the results were not reported for treatment groups separately. Molteni et al. [92] used the POSAS to evaluate donor sites and found FTSG performing better than STSG when assessed by both patient and observer. Peters et al. [91] used an optical three-dimensional scanner to objectively compare skin grafts at the donor site and noticed a significantly greater surface deviation in the STSG group. Selvaggi et al. [58] performed a subjective evaluation of the aesthetic outcome using a survey, but without statistical testing. Thiele et al. [93] observed more hypertrophic scar formation in the FTSG group than in the STSG group, but without testing for significancy. Vahldieck et al. [23] evaluated donor sites using the POSAS [83]. With respect to the clinician-based outcome, the authors observed a significantly better outcome in terms of scar relief, pigmentation, and overall impression and a significantly better overall score, all in favor of the FTSG treatment group. In contrast, the patient-based aesthetic outcomes were not significantly different. Zuidam et al. [16] used the VSS and also a visual analog score to assess the aesthetic outcome. While the visual analog score was similar for both treatment groups, the VSS showed a significantly improved outcome for pliability in favor of the FTSG group.

### Meta-analysis

#### Major wound complications

**Fig. 5:**
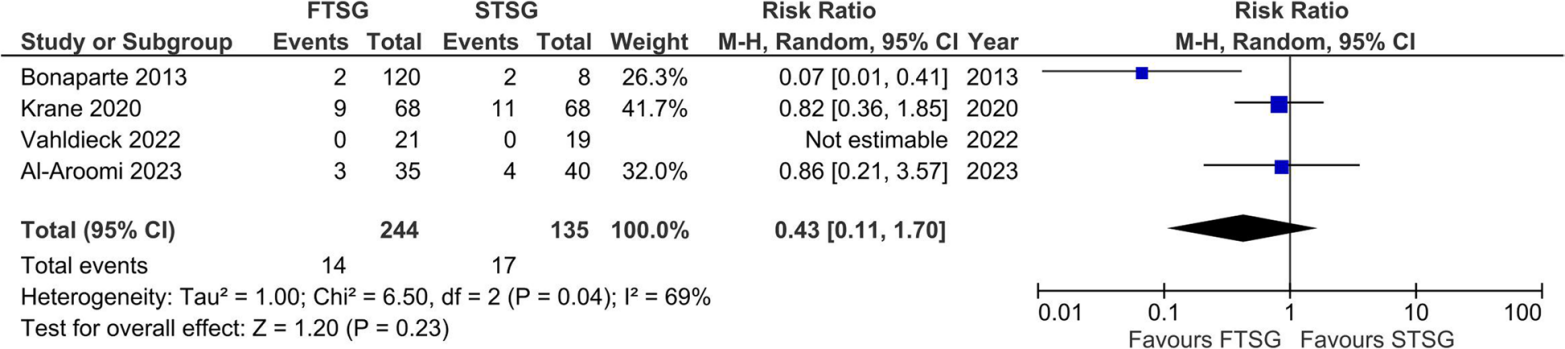
Forest plot of major wound complications

Four studies with a total of 244 donor site closures using FTSG and 135 donor site closures using STSG were included in the meta-analysis [23, 90, 94, 96]. A high level of heterogeneity was measured (*I*^2^ = 69%), and a random-effects model to calculate the pooled effect size detected no statistically significant differences in occurrence of major wound complications between groups (*RR* 0.43; 95% *CI* 0.11 to 1.70; *p* = 0.23) (Fig. 5).

To assess the potential influence of study design on the pooled outcomes, we performed a sensitivity analysis including only retrospective studies [23, 90, 96]. The level of heterogeneity decreased substantially (*I*^2^ = 0%), and the pooled effect estimate shifted closer to no difference (*RR* 0.83; 95% *CI* 0.41 to 1.68; *p* = 0.60), again showing no statistically significant difference between groups (Additional file 5).

#### Minor wound complications

**Fig. 6:**
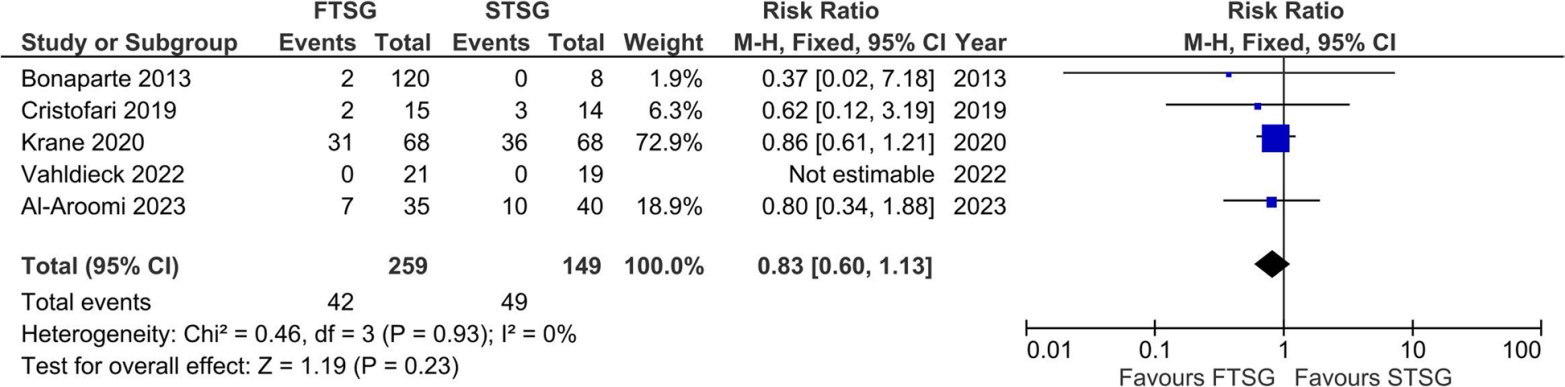
Forest plot of minor wound complications

Five studies with a total of 259 donor site closures using FTSG and 149 donor site closures using STSG were included in the meta-analysis [23, 85, 90, 94, 96]. A low level of heterogeneity was measured (*I*^2^ = 0%), and a fixed-effects model to calculate the pooled effect size showed no statistically significant differences in occurrence of minor wound complications between groups (*RR* 0.83; 95% *CI* 0.60 to 1.13; *p* = 0.23) (Fig. 6).

Sensitivity analysis including only retrospective studies was performed [23, 85, 90, 96]. Again, a low level of heterogeneity was measured (*I*^2^ = 0%), and a fixed-effects model showed no statistically significant differences between groups (*RR* 0.83; 95% *CI* 0.61 to 1.15; *p* = 0.26) (Additional file 6).

#### Data not amenable to meta-analysis

Cristofari et al. [85] and Vahldieck et al. [23] reported on both function-related outcomes and aesthetics-related outcomes, but given the strong heterogeneity in the assessment scales used (DASH vs. ROM in functional assessment and VSS and a customized satisfaction scale vs. POSAS in aesthetic assessment), meta-analysis was not feasible.

### Certainty of evidence

The evidence was graded at a low or very low certainty with reasons for downgrading including risk of bias, imprecision, and inconsistency (Additional file 2). The evidence for major wound complications was downgraded to very low certainty (once for risk of bias, once for inconsistency, and once for imprecision), and the evidence for minor wound complications was downgraded to low certainty (once for risk of bias and once for imprecision).

## Discussion

With significant improvements in microvascular reconstruction techniques, our focus has shifted to reducing donor site complications. At the same time, our patients expect optimal therapy. Therefore, the choice of closure technique should ideally be based on evidence rather than personal preference. However, after 30 years of ongoing discussion [6], we still do not know whether FTSG or STSG should be preferred for RFFF donor site closure.

This is the first systematic review solely focusing on fasciocutaneous RFFF that has been conducted on this topic, with a published study protocol, a thorough risk-of-bias assessment, and a certainty of evidence assessment using the GRADE approach. We included 15 studies with a total of 933 procedures, comparing RFFF donor site closure using FTSG vs. STSG. Our meta-analysis regarding the incidence of minor and major donor site complications showed no significant differences between treatment groups.

While earlier studies, such as those by Mosquera et al. [68], Saleki et al. [67], and Zhang et al. [69], have suggested differing outcomes, our findings suggest that the choice between FTSG and STSG may not have a universally superior option in terms of clinical outcomes. This highlights the need for clinicians to consider factors beyond universal graft choice, such as patient-specific factors (e.g., skin tone or skin quality), surgical factors (e.g., surgical expertise and surgical planning) and donor site characteristics (e.g., defect size and tissue availability) when planning RFFF surgery.

The meta-analysis of major wound complications revealed a high-level heterogeneity among studies. (*I*^2^ = 69%, *χ*^2^ = 6.50, *p* = 0.04) (Fig. 5). Sensitivity analysis including only retrospective cohort studies showed a pooled effect closer to the null, along with the reduction in heterogeneity. This highlights the influence of Bonaparte et al.’s study (*n* = 177) in which FTSG closure clearly outperformed STSG closure [94]. The authors conducted a three-armed study using DynaClose (Canica Design Inc., Montreal, Canada), a system for preoperative skin expansion using elastic tape that was adhered to the skin under tension 2 weeks preoperatively. The three treatment groups were as follows: primary closure, closure by local FTSG, and closure by distant STSG. Primary closure of the donor site was always attempted. If there were still areas of the wound with insufficient skin for primary closure, a local FTSG was harvested from redundant skin on the ipsilateral forearm incision line. When this was also insufficient, a distant STSG was utilized to close the defect. Participants in the STSG group were all patients in which skin expansion failed because of nonadherence to the protocol or in cases where tension was found to be insufficient. The latter occurred mainly in young, otherwise healthy males. STSG showed a higher complication rate (25% major wound complications) compared to primary closure (0% major wound complications) and local FTSG (1.7% major wound complications), even though donor site defect sizes were similar between groups. It could be hypothesized that an FTSG simply yields a better result than an STSG for anatomical reasons, but this is not supported by other studies in our meta-analysis [90, 94, 96]. Another hypothesis is that preoperative skin expansion has a positive effect on tissue viability. The latter is supported by research on histophysiological changes during controlled skin expansion [105, 106]. During skin expansion, a variety of histologic changes are observed, including increased epidermal mitotic activity and increased vascularity in expanded tissue. A study by Cherry et al. [107] on the vascularity and survival of skin flaps in controlled, expanded pig skin revealed increased vascularity on angiograms along with a 117% increase in survival length compared to nonexpanded skin flaps. These histological changes, initiated by the preoperative expansion, might have contributed to the lower complication rate in the treatment group that underwent successful pre-expansion. Although these data originated from an animal study, it should be noted that generally, both human and animal soft tissues exhibit similar responses during controlled tissue expansion [105]. The increased vascularity observed could stem from various factors such as vessel realignment, arteriovenous shunt closure, angiogenesis, and a decrease in neurohumoral vasoactive agents [106, 108]. Preoperative skin expansion is an affordable and intuitive solution that could significantly improve the likelihood of primary wound closure [94]. When primary wound closure is not feasible, it could still increase the viability of the locally harvested FTSG, potentially reducing major wound complications such as tendon exposure and complete graft loss. The suggestion that preoperative skin expansion could improve graft survival, coupled with the lack of clear evidence of superiority between FTSG and STSG, justifies further research.

The high heterogeneity in outcomes for major wound complications contrasts with the low heterogeneity in minor wound complications (*I*^2^ = 0%) (Fig. 6). This contrast is remarkable, given the high overlap among studies in both analyses. In fact, all four studies included in the major donor site complications meta-analysis were also included in the minor donor site complications meta-analysis plus one extra study. Minor complications are probably less influenced by the surgical technique (e.g., preoperative skin expansion), which affects major complications. However, this discrepancy can be explained by the fact that preoperative skin expansion does not necessarily lead to a reduction in minor wound complications like infection, hematoma, or seroma.

When evaluating strategies to reduce donor site morbidity, the flap raising technique may also be important. Some researchers advocate the suprafascial dissection technique, suggesting it results in lower morbidity, as the deep forearm fascia is preserved [100]. This not only offers a vascularized bed suitable for grafting but also maintains a protective connective tissue layer over the tendons. A comprehensive comparison between suprafascial and subfascial techniques is beyond the scope of this study, but authors utilizing suprafascial dissection indeed report few or even zero major donor site complications, such as complete skin graft loss (0–6%) and tendon exposure (0–4%) [16, 23, 30, 84, 93, 100]. The establishment of future controlled studies to further explore these findings would be beneficial.

With respect to functional outcome, FTSG was thought to lead to better results compared to STSG, as it includes both epidermis and the full dermis layer [109]. The thicker FTSG would provide greater elasticity and durability and could therefore better withstand the mechanical stress that occurs in highly flexible regions such as the wrist. During wound healing, FTSGs shows a lower degree of graft contraction compared to STSGs [110], which would be good for preserving range of motion. On the other hand, one might reason that an STSG would yield better results since it generally heals faster and the metabolic needs are lower, due to its thinner structure [109]. Despite these theoretical considerations, hardly any difference was detected when comparing FTSG to STSG. One study favored FTSG [85], one study favored STSG [97], and all other studies did not reveal significant differences [16, 23, 90, 92], observed mixed results [96], or did not undertake statistical analysis [13, 58, 84, 93]. For aesthetic outcome, wound closure with FTSG was thought to yield better results as more characteristics of the donor skin are preserved, as the thicker FTSG contains more collagen, dermal vascular plexuses, and epithelial appendages [109]. It could therefore provide a better match in skin color and texture, resulting in a more natural appearance [109, 111]. Most of the included studies support this hypothesis as seven studies favored FTSG [16, 23, 90–92, 95, 96], no studies favored STSG, and two studies observed no statistical differences [85, 97]. Four studies did not undertake statistical analysis [13, 58, 84, 93]. Since the available data were not amenable to meta-analysis, definitive conclusions cannot be drawn regarding functional and aesthetic outcome.

Limitations of this study are the relatively high risk of bias in the included studies, the inconsistency, the imprecision, and therefore the low to very low certainty of evidence. To increase the certainty of evidence, well-designed RCTs are needed. In addition, the majority of the included studies were retrospective in nature. After removing the only prospective study, the pooled effect estimate shifted closer to no difference. Therefore, more prospective studies are needed to further explore the validity of the outcomes of the current meta-analysis. Researchers should report variables such as defect size and time-to-follow-up. In instances where significant differences in these patient characteristics are observed between groups, adjusting for these discrepancies is crucial to ensure the validity of the findings. As this systematic review is designed as a living document, we plan to update when new evidence becomes available.

## Conclusion

This systematic review and meta-analysis showed no conclusive evidence of a difference in wound outcome between RFFF donor site closure with FTSG versus STSG. These conclusions are based on data from five studies with a low to very low certainty according to GRADE and should be interpreted cautiously. To define the potential surgical impact of utilizing FTSG vs. STSG more clearly and to increase the certainty of the evidence, further research is needed. We suggest conducting RCTs, designed in line with the Standard Protocol Items: Recommendations for Interventional Trials (SPIRIT) statement [112] and reported according to the Consolidated Standards of Reporting Trials (CONSORT) statement [113]. Until more robust evidence becomes available, the optimal skin graft choice should be guided by patient-specific factors, surgical considerations, and donor site characteristics.

## Supplementary Information

Additional file 1: Search strategy.docx.

Additional file 2: GRADE evidence & summary table.docx.

Additional file 3: Citationchaser URL.docx.

Additional file 4: PRISMA 2020 checklist.docx.

Additional file 5: Forest plot - major wound complication (retrospective only).jpg.

Additional file 6: Forest plot - minor wound complication (retrospective only).jpg.

## Data Availability

Not applicable.

## Abbreviations

CENTRAL: Cochrane Central Register of Controlled Trials
CISS: Cold Intolerance Severity Score
CNKI: China National Knowledge Infrastructure
CONSORT: Consolidated Standards of Reporting Trials
DASH: Disability of Arm, Shoulder and Hand
FTSG: Full-thickness skin graft
GRADE: Grading of Recommendations Assessment, Development and Evaluation
OCRFFF: Osteocutaneous radial forearm free flap
PICO: Patient, intervention, comparison, and outcome
POSAS: Patient and Observer Scar Assessment Scale
PRISMA: Preferred Reporting Items for Systematic reviews and Meta-Analyses
PROSPERO: International Prospective Register of Systematic Reviews
RCT: Randomized controlled trial
RFFF: Radial forearm free flap
RoB 2: Cochrane Risk-of-Bias tool version 2
ROBINS-I: Risk Of Bias In Non-Randomized Studies—of Interventions
ROM: Range of motion
RR: Risk ratio
SoF: Summary of findings
SPIRIT: Standard Protocol Items: Recommendations for Interventional Trials
STSG: Split-thickness skin graft
VSS: Vancouver Scar Scale
