# Supplementary material for: Full-thickness skin graft versus split-thickness skin graft for fasciocutaneous radial forearm free flap donor site closure: a systematic review and meta-analysis
Source: Syst Rev. 2025 May 27;14:118. doi: 10.1186/s13643-025-02863-7 (PMC12108030; doi:10.1186/s13643-025-02863-7)
Supplement: Supplementary file 2 — Additional file 2: GRADE evidence & summary table.docx. [file 13643_2025_2863_MOESM2_ESM.docx]

**Grade Evidence profile:**

**Question:** FTSG compared to STSG for radial forearm free flap donor-site closure

| **Certainty assessment** | | | | | | | **№ of patients** | | **Effect** | | **Certainty** | **Importance** |
| --- | --- | --- | --- | --- | --- | --- | --- | --- | --- | --- | --- | --- |
| **№ of studies** | **Study design** | **Risk of bias** | **Inconsistency** | **Indirectness** | **Imprecision** | **Other considerations** | **FTSG** | **STSG** | **Relative (95% CI)** | **Absolute (95% CI)** |  |  |
| **Major wound complications** | | | | | | | | | | | | |
| 4 | non-randomised studies | serious | serious^a^ | not serious | serious^b^ | none | 14/244 (5.7%) | 17/135 (12.6%) | **RR 0.43** (0.11 to 1.70) | **72 fewer per 1.000** (from 112 fewer to 88 more) | ⨁◯◯◯ Very low^a,b^ |  |
| **Minor wound complications** | | | | | | | | | | | | |
| 5 | non-randomised studies | serious | not serious | not serious | serious^b^ | none | 42/259 (16.2%) | 49/149 (32.9%) | **RR 0.83** (0.60 to 1.13) | **56 fewer per 1.000** (from 132 fewer to 43 more) | ⨁⨁◯◯ Low^b^ |  |

**CI:** confidence interval; **RR:** risk ratio

#### Explanations

a. High heterogeneity.

b. Optimal Information Size (OIS) not met.

| **Summary of findings:** | | | | | | |
| --- | --- | --- | --- | --- | --- | --- |
| **FTSG compared to STSG for radial forearm free flap donor-site closure** | | | | | | |
| **Patient or population:** patients with tissue defects in the maxillofacial, limb or genital region requiring reconstruction with a fasciocutaneous RFFF  **Intervention:** FTSG  **Comparison:** STSG | | | | | | |
| Outcomes | **Anticipated absolute effects^*^** (95% CI) | | Relative effect (95% CI) | № of participants (studies) | Certainty of the evidence (GRADE) | Comments |
|  | **Risk with STSG** | **Risk with FTSG** |  |  |  |  |
| Major wound complications | 126 per 1.000 | **54 per 1.000** (14 to 214) | **RR 0.43** (0.11 to 1.70) | 379 (4 non-randomised studies) | ⨁◯◯◯ Very low^a,b^ |  |
| Minor wound complications | 329 per 1.000 | **273 per 1.000** (197 to 372) | **RR 0.83** (0.60 to 1.13) | 408 (5 non-randomised studies) | ⨁⨁◯◯ Low^b^ |  |
| ***The risk in the intervention group** (and its 95% confidence interval) is based on the assumed risk in the comparison group and the **relative effect** of the intervention (and its 95% CI).  **CI:** confidence interval; **RR:** risk ratio | | | | | | |
| **GRADE Working Group grades of evidence** **High certainty:** we are very confident that the true effect lies close to that of the estimate of the effect. **Moderate certainty:** we are moderately confident in the effect estimate: the true effect is likely to be close to the estimate of the effect, but there is a possibility that it is substantially different. **Low certainty:** our confidence in the effect estimate is limited: the true effect may be substantially different from the estimate of the effect. **Very low certainty:** we have very little confidence in the effect estimate: the true effect is likely to be substantially different from the estimate of effect. | | | | | | |

#### Explanations

a. High heterogeneity.

b. Optimal Information Size (OIS) not met.
