# Supplementary material for: Full-thickness skin graft versus split-thickness skin graft for fasciocutaneous radial forearm free flap donor site closure: a systematic review and meta-analysis
Source: Syst Rev. 2025 May 27;14:118. doi: 10.1186/s13643-025-02863-7 (PMC12108030; doi:10.1186/s13643-025-02863-7)
Supplement: Supplementary file 3 — Additional file 3: Citationchaser URL.docx. [file 13643_2025_2863_MOESM3_ESM.docx]

<https://estech.shinyapps.io/citationchaser/?dois=10.1016/j.ijom.2023.04.003,10.1016/j.bjoms.2007.04.008,10.1016/j.oooo.2013.01.007,10.1016/s1010-5182(97)80060-1,10.1016/j.anplas.2019.06.009,10.1016/j.otohns.2005.09.019,10.1177/0194599819901124,10.1097/00006534-199901000-00021,10.1007/s00238-021-01922-1,10.1097/SCS.0000000000007820,10.1097/01.prs.0000221110.43002.a0,10.1007/s00238-008-0238-y,10.1016/j.jcms.2022.06.010,10.1097/01.sap.0000185656.66239.ad,10.3342/kjorl-hns.2019.00129>
