# Supplementary figures and images for: Full-thickness skin graft versus split-thickness skin graft for fasciocutaneous radial forearm free flap donor site closure: a systematic review and meta-analysis
Source: Syst Rev. 2025 May 27;14:118. doi: 10.1186/s13643-025-02863-7 (PMC12108030; doi:10.1186/s13643-025-02863-7)

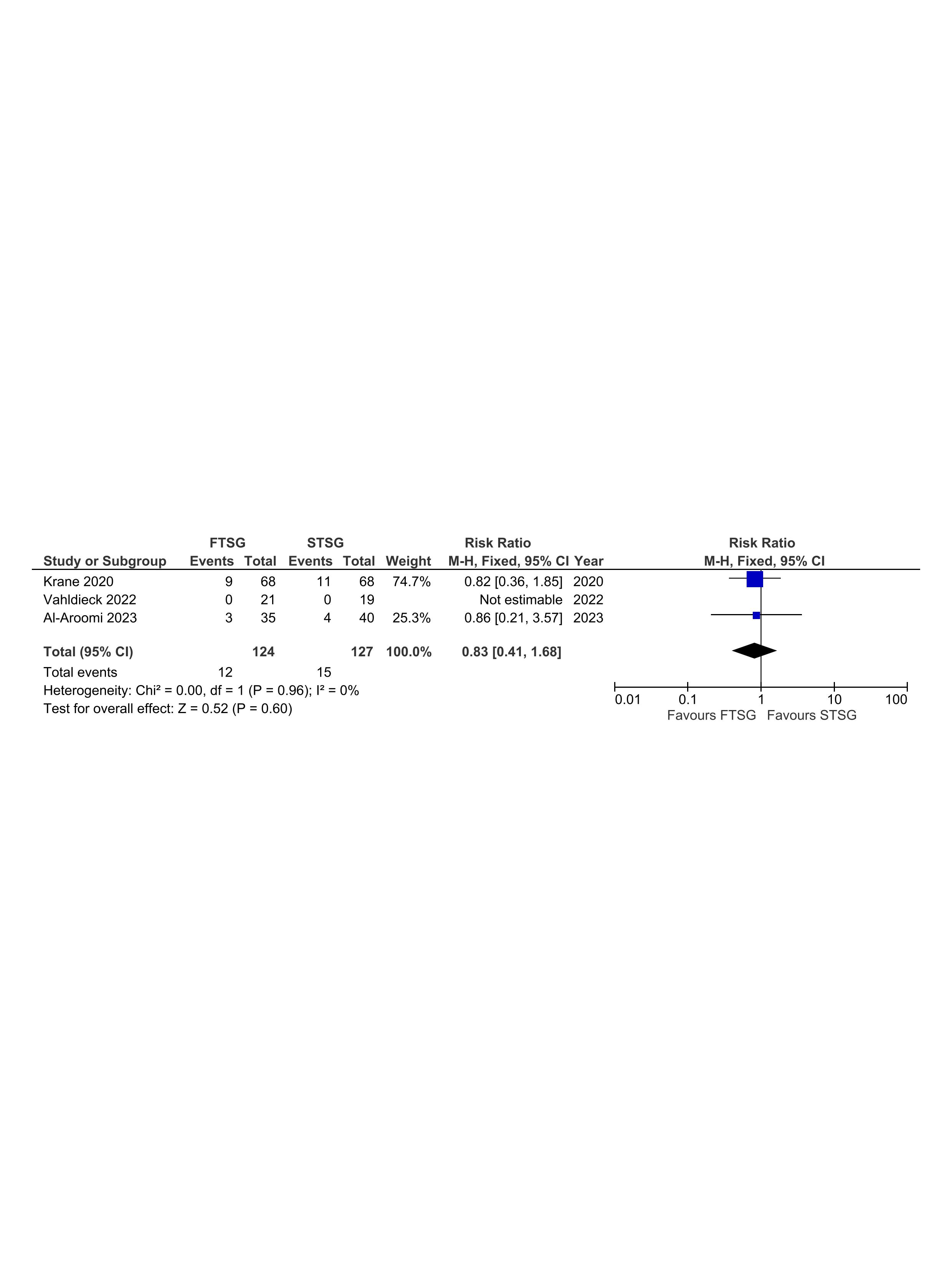

Supplement: Supplementary file 5 — Additional file 5: Forest plot - major wound complication (retrospective only).jpg. [file 13643_2025_2863_MOESM5_ESM.jpg]

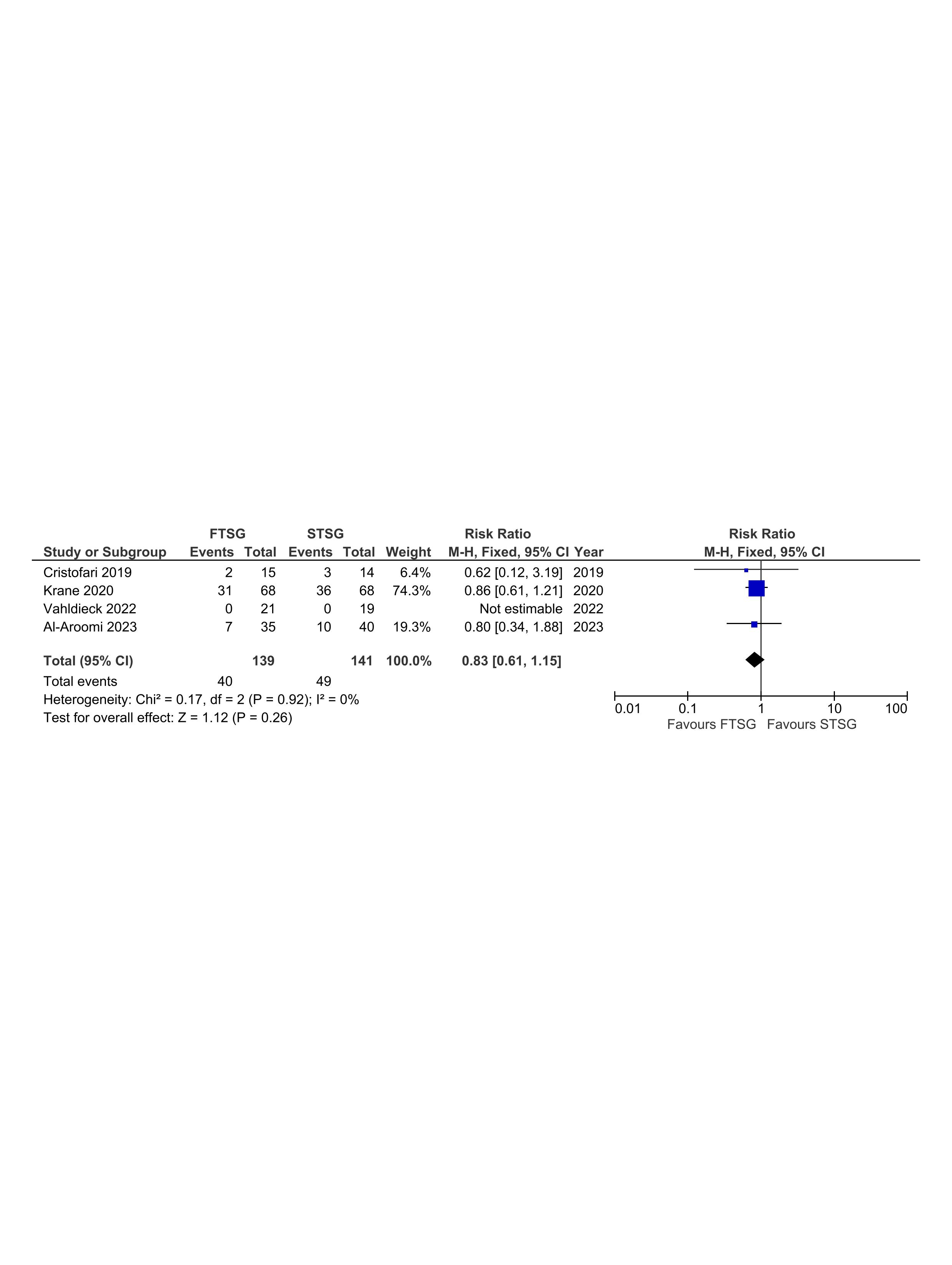

Supplement: Supplementary file 6 — Additional file 6: Forest plot - minor wound complication (retrospective only).jpg. [file 13643_2025_2863_MOESM6_ESM.jpg]
